# Supplementary material for: A chloroplast retrograde signal, 3’-phosphoadenosine 5’-phosphate, acts as a secondary messenger in abscisic acid signaling in stomatal closure and germination
Source: eLife. 2017 Mar 21;6:e23361. doi: 10.7554/eLife.23361 (PMC5406205; doi:10.7554/eLife.23361)
Supplement: Supplementary file 2: — DOI: http://dx.doi.org/10.7554/eLife.23361.020 [file elife-23361-supp2.docx]

**Supplementary File 2: List of ABA signaling genes transcriptionally altered in *ost1*-2 *sal1*-8 +/- ABA.**

| **Complementation category** | **Type of transcriptional regulation** |
| --- | --- |
| **1** | Constitutive expression of ABA signaling genes in *ost1*-2 *sal1*-8 |
| **2** | ABA-induced mis-expression specifically in *ost1*-2 *sal1*-8, but not in WT |

| **Category** | **Gene** | **Clade / Group** | **AGI** | **Fold Change** | | | **Role(s) in ABA signaling** | **Ref** | **Expression pattern**  **(from eFP Browser) (**[***1***](#_ENREF_1)**)** | **Cat.** |
| --- | --- | --- | --- | --- | --- | --- | --- | --- | --- | --- |
|  |  |  |  | **ost1-2 alx8** | **ost1-2 alx8 +ABA** | **WT +ABA** |  |  |  |  |
| Transcription factor | MYC2 | - | AT1G32640 | - | 2.71 | - | Transcriptional Regulation | ([*2*](#_ENREF_2)) | Expressed in root, shoot and flowers. Induced in root in response to multiple abiotic stresses. | 2 |
|  | MYB61 | - | AT1G09540 | 0.61 | - | - | Transcriptional Regulation | ([*3*](#_ENREF_3)) | Almost constant expression in all tissue types | 1 |
| Calcium Signaling | Calcium-dependent protein kinase (CDPK)-related kinase 2 (CRK2) | - | AT3G19100 | 2.08 | 3.58 | 3.53 | Role unknown | - | Expressed in most tissues. Induced by osmotic shock and salt stress | 1 |
|  | CDPK-related kinase 8 (CRK8) | - | AT1G49580 | - | 1.98 | - | Role unknown | - | Low in most tissues, highest in developing seed. Downregulated by abiotic stress. | 2 |
|  | Calcium-dependent protein kinase 34 (CDPK34) | II | AT5G19360 | 3.45 | 4.59 | 1.60 | Unknown, but related to group II CDPKs (CDPK3, CDPK21, CDPK23) involved in regulation of anion channels SLAC1 and SLAH3 for stomatal closure | ([*4*](#_ENREF_4)*,* [*5*](#_ENREF_5)) | Undetectable under standard conditions. Induced by multiple abiotic stresses, ABA | 1 |
|  | CDPK9 | II | AT5G23580 | 1.71 | 1.90 | - |  |  | Expressed in multiple tissues. Expression is not induced by stress or ABA. | 1 |
|  | CDPK19 | II | AT5G19450 | - | 1.54 | - | Regulates Catalase 3 (CAT3). KO has lower catalase activity, higher ROS, and diminished calcium inhibition of potassium inward currents. | ([*6*](#_ENREF_6)) | Strong expression in most tissues, but unchanged in response to ABA. Slightly induced in roots in response to osmotic shock | 2 |
|  | CDPK30 | III | AT1G74740 | 0.53 | - | - | Binds to ABA signaling transcription factor ABF4 | ([*7*](#_ENREF_7)) | Weak in all tissues. Not induced by stress or ABA. | 1 |
|  | CDPK32 | III | AT3G57530 | 2.25 | 2.77 | 1.75 | Regulates ABF4 activity and ABF4-regulated genes. Also binds to TFs (ABF1, ABF2, and ABF3). | ([*7*](#_ENREF_7)) | Strong in pollen, almost none in other tissues. Expression is not induced by abiotic stresses | 1 |
|  | CDPK28 | IV | AT5G66210 | 2.05 | 2.32 | - | Role(s) unknown. Unlike group III CDPKs, CPK28 does not interact with ABF4 | ([*7*](#_ENREF_7)) | Strong in leaves. Not induced by ABA, but is induced in root in response to salt | 1 |
|  | Calcineurin B-like Protein 7 (CBL7) | - | AT4G26560 | 2.17 | 3.01 | - | Unknown. Related to CBL2 and CBL3 which are calcium sensors important for ABA responses. Similar to CBL2 and CBL3, CBL6 and CBL7 also contain tonoplast targeting sequences. | ([*8-10*](#_ENREF_8)) | Weak in most tissues. Expression is not strongly changed in response to abiotic stress or ABA | 1 |
|  | CBL6 | - | AT4G16350 | 0.54 | 0.51 | 0.46 |  |  | Weakly expressed in most tissues. Expression is slightly downregulated by abiotic stresses. | 1 |
|  | CBL-interacting serine/threonine-protein kinase 6 (CIPK6) | I | AT4G30960 | 1.76 | 3.18 | 2.75 | Activates the key K+ channel in ABA signaling, AKT1. Interacts with ABA signaling regulator CBL1, which regulates ROS and NADPH oxidase activity. | ([*11-13*](#_ENREF_11)) | Moderately expressed in most tissues. Expression induced by osmotic shock, salt, drought and ABA. | 1 |
|  | CIPK10 | I | AT5G58380 | 1.60 | 1.60 | - | Unknown. Interacts with CBL9, which regulates ROS homeostasis and NADPH oxidase. Closely related to CIPK15 involved in ABA signaling. | ([*13*](#_ENREF_13)*,* [*14*](#_ENREF_14)) | Strong in pollen, almost none in other tissues. Induced by osmotic shock and salt stress. | 1 |
|  | CIPK7 | I | AT3G23000 | - | 1.97 | - | Unknown. Binds CBL1, which regulates ROS homeostasis in ABA signaling and stomatal closure | ([*13*](#_ENREF_13)) | Weakly expressed in most tissues. Expression induced by cold, osmotic shock and salt. | 2 |
|  | CIPK1 | II | AT3G17510 | 2.14 | 1.79 | 0.65 | Regulator of ABA responses. Binds the ABA signaling regulator CBL1. | ([*12*](#_ENREF_12)*,* [*13*](#_ENREF_13)) | Strong in pollen, very low in other tissues. Induced in shoots, roots by osmotic shock, drought, salt. | 1 |
|  | CIPK21 | II | AT5G57630 | 0.43 | 0.46 | - | Interacts with CBL1, 2, 3 and 9, which regulate ABA signaling. Closely related to CIPK1, a regulator of ABA responses. | ([*12*](#_ENREF_12)*,* [*13*](#_ENREF_13)*,* [*15*](#_ENREF_15)) | Moderately expressed in most tissues, strongest in pollen. Induced in root and shoot by cold, osmotic shock and drought, but not ABA. | 1 |
|  | CIPK3 | II | AT2G26980 | - | 0.63 | - | Related to CIPK26, regulator of ABA responses that interacts with CBL9, which regulates ROS and NADPH oxidase activity | ([*13*](#_ENREF_13)*,* [*16*](#_ENREF_16)) | Strongly expressed in mature leaves. Expression slightly downregulated by abiotic stress and ABA. | 2 |
| ROS Homeostasis | Glutathione Peroxidase 3 (GPX3) | - | AT2G43350 | 0.63 | 0.56 | - | Negative regulator of ROS. KOs have increased ROS and stomatal closure in response to ABA | ([*17*](#_ENREF_17)) | Strongly expressed in most tissues. Expression slightly downregulated by abiotic stresses. | 1 |
| SNARE Complexes / Protein Import | Syntaxin 41 (SYP41) | 4 | AT5G26980 | - | 2.36 | - | Specific role unknown, but forms protein complex with SYP61, which is important for stomatal closure | ([*18*](#_ENREF_18)*,* [*19*](#_ENREF_19)) | Moderate in most tissues. Induced in root and shoot by osmotic shock and drought, not by ABA. | 2 |
|  | Syntaxin 124 (SYP124) | 12 | AT1G61290 | 2.04 | 1.95 | - | Unknown, but related to SYP121, which regulates stomatal closure together with SYP61 | ([*19*](#_ENREF_19)*,* [*20*](#_ENREF_20)) | Strong in pollen, almost none in other tissues. Expression induced in roots by salt stress. | 1 |

**Supplementary References**

1. D. Winter *et al.*, An "Electronic Fluorescent Pictograph" browser for exploring and analyzing large-scale biological data sets. *PLoS One* **2**, e718 (2007).

2. H. Abe *et al.*, Arabidopsis AtMYC2 (bHLH) and AtMYB2 (MYB) Function as Transcriptional Activators in Abscisic Acid Signaling. *Plant Cell* **15**, 63 (2003).

3. Y. K. Liang *et al.*, AtMYB61, an R2R3-MYB transcription factor controlling stomatal aperture in Arabidopsis thaliana. *Curr Biol* **15**, 1201 (Jul 12, 2005).

4. S. Cheng, M. R. Willmann, H. Chen, J. Sheen, Calcium Signaling through Protein Kinases. The Arabidopsis Calcium-Dependent Protein Kinase Gene Family. *Plant Physiology* **129**, 469 (2002).

5. D. Geiger *et al.*, Guard cell anion channel SLAC1 is regulated by CDPK protein kinases with distinct Ca(2+) affinities. *Proceedings of the National Academy of Sciences of the United States of America* **107**, 8023 (2010).

6. J.-J. Zou *et al.*, Arabidopsis CALCIUM-DEPENDENT PROTEIN KINASE8 and CATALASE3 Function in Abscisic Acid-Mediated Signaling and H2O2 Homeostasis in Stomatal Guard Cells under Drought Stress. *The Plant Cell*, (May 12, 2015, 2015).

7. H.-i. Choi *et al.*, Arabidopsis Calcium-Dependent Protein Kinase AtCPK32 Interacts with ABF4, a Transcriptional Regulator of Abscisic Acid-Responsive Gene Expression, and Modulates Its Activity. *Plant Physiology* **139**, 1750 (2005).

8. Ü. Kolukisaoglu, S. Weinl, D. Blazevic, O. Batistic, J. Kudla, Calcium Sensors and Their Interacting Protein Kinases: Genomics of the Arabidopsis and Rice CBL-CIPK Signaling Networks. *Plant Physiology* **134**, 43 (2004).

9. O. Batistič *et al.*, S-acylation-dependent association of the calcium sensor CBL2 with the vacuolar membrane is essential for proper abscisic acid responses. *Cell Research* **22**, 1155 (2012).

10. T. Kleist, A. Spencley, S. Luan, Comparative Phylogenomics of the CBL-CIPK Calcium-decoding Network in the Moss Physcomitrella, Arabidopsis, and Other Green Lineages. *Frontiers in Plant Science* **5**, (2014-May-14, 2014).

11. W.-Z. Lan, S.-C. Lee, Y.-F. Che, Y.-Q. Jiang, S. Luan, Mechanistic Analysis of AKT1 Regulation by the CBL–CIPK–PP2CA Interactions. *Molecular Plant* **4**, 527 (2011).

12. C. D'Angelo *et al.*, Alternative complex formation of the Ca2+-regulated protein kinase CIPK1 controls abscisic acid-dependent and independent stress responses in Arabidopsis. *The Plant Journal* **48**, 857 (2006).

13. M. M. Drerup *et al.*, The Calcineurin B-Like Calcium Sensors CBL1 and CBL9 Together with Their Interacting Protein Kinase CIPK26 Regulate the Arabidopsis NADPH Oxidase RBOHF. *Molecular Plant* **6**, 559 (2013).

14. Y. Guo *et al.*, A Calcium Sensor and Its Interacting Protein Kinase Are Global Regulators of Abscisic Acid Signaling in Arabidopsis. *Developmental Cell* **3**, 233 (2002).

15. G. K. Pandey *et al.*, CBL-interacting protein kinase, CIPK21, regulates osmotic and salt stress responses in Arabidopsis. *Plant Physiol*, (Jul 21, 2015).

16. G. K. Pandey *et al.*, Calcineurin-B-Like Protein CBL9 Interacts with Target Kinase CIPK3 in the Regulation of ABA Response in Seed Germination. *Molecular Plant* **1**, 238 (2008).

17. Y. Miao *et al.*, An Arabidopsis glutathione peroxidase functions as both a redox transducer and a scavenger in abscisic acid and drought stress responses. *The Plant Cell* **18**, 2749 (Oct, 2006).

18. J. Zhu *et al.*, OSM1/SYP61: a syntaxin protein in Arabidopsis controls abscisic acid-mediated and non-abscisic acid-mediated responses to abiotic stress. *The Plant Cell* **14**, 3009 (Dec, 2002).

19. A. A. Sanderfoot, V. Kovaleva, D. C. Bassham, N. V. Raikhel, Interactions between syntaxins identify at least five SNARE complexes within the Golgi/prevacuolar system of the Arabidopsis cell. *Mol Biol Cell* **12**, 3733 (Dec, 2001).

20. C. Eisenach, Z. H. Chen, C. Grefen, M. R. Blatt, The trafficking protein SYP121 of Arabidopsis connects programmed stomatal closure and K(+) channel activity with vegetative growth. *Plant J* **69**, 241 (Jan, 2012).
